# Comparing Shor and Steane error correction using the Bacon-Shor code

**DOI:** 10.1126/sciadv.adp2008

**Published:** 2024-11-06

**Authors:** Shilin Huang, Kenneth R. Brown, Marko Cetina

**Affiliations:** ^1^ Duke Quantum Center, Duke University, Durham, NC 27701, USA.; ^2^ Department of Electrical and Computer Engineering, Duke University, Durham, NC 27708, USA.; ^3^ Department of Physics, Duke University, Durham, NC 27708, USA.; ^4^ Department of Chemistry, Duke University, Durham, NC 27708, USA.

## Abstract

Quantum states decohere through interaction with the environment. Quantum error correction can preserve coherence through active feedback wherein quantum information is encoded into a logical state with a high degree of symmetry. Perturbations are detected by measuring the symmetries of the state and corrected by applying gates based on these measurements. To measure the symmetries without perturbing the data, ancillary quantum states are required. Shor error correction uses a separate quantum state for the measurement of each symmetry. Steane error correction maps the perturbations onto a logical ancilla qubit, which is then measured to check several symmetries simultaneously. We experimentally compare Shor and Steane correction of bit flip errors using the Bacon-Shor code implemented in a chain of 23 trapped atomic ions. We find that the Steane method produces fewer errors after a single round of error correction and less disturbance to the data qubits without error correction.

## Teaser

Steane error correction outperforms the Shor approach on a fully connected trapped-ion platform.

## INTRODUCTION

Quantum computers promise to solve computationally difficult cryptographic and chemistry problems using billions or trillions of quantum gates (*1*, *2*). Current quantum devices are limited to hundreds of quantum gates, and many believe that the path forward is quantum error correction (EC), which enables us to make reliable qubits and gates out of unreliable components.

In the past few years, multiple experiments have demonstrated key elements of quantum EC. Highlights include improved state preparation with lower measurement error (*3*, *4*), extended coherence times (*5*), transversal logical gates (*3*, *6*–*8*), repeated rounds of syndrome extraction (*6*, *9*–*12*), magic-state injection (*8*), improved performance with code distance (*11*), and encoded circuits with multiple logical qubits (*13*). While no experiment has demonstrated an improvement in all aspects of an encoded qubit relative to its physical counterpart, these early works tested our assumptions about quantum EC protocols and the underlying noise models. These tests help us chart the path toward useful and scalable quantum EC.

Quantum EC encodes logical qubits into multiple physical qubits. The encoded code space has a number of symmetries whose actions are changed by the detectable errors. Stabilizer codes have binary symmetries corresponding to the +1 and −1 eigenvalues of the stabilizer operators, which form a commuting group of *n*-qubit Pauli operators (*14*). We measure the eigenvalues of a subset of the stabilizers that generates the whole code space. The error syndrome is a bit string where the 1’s indicate that the corresponding stabilizer generator has an eigenvalue that is opposite of that of the code space.

A critical part of a quantum EC procedure is the fault-tolerant measurement of the error syndrome. We use the term Shor EC for all approaches that measure the stabilizer generators of the error syndrome one at a time. Traditionally, this requires the preparation of an entangled cat state as an encoded ancilla qubit for each generator (*15*). For some codes, a single qubit is an effective ancilla qubit (*16*–*18*), and for any code, a flag qubit system can be used instead of entangled ancilla qubits (*19*). Knill and Steane present alternative methods where logical qubits in the same code are used to determine the error syndrome in one or two steps (*20*, *21*). Many other methods are possible (*22*), especially when considering the interplay between noisy syndrome bits and repeated measurements (*23*).

**Fig. 1.**
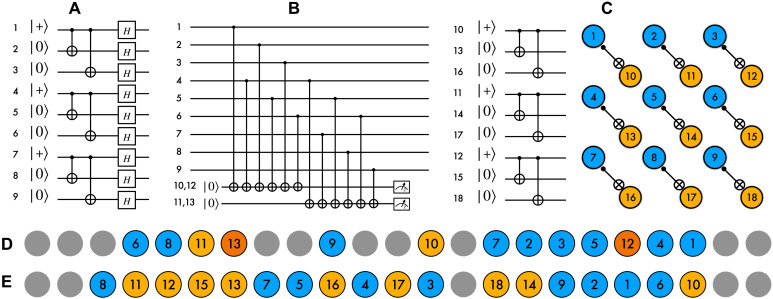
Implementation of Shor- and Steane-style error checks of the Bacon-Shor code using 23 trapped ions. (**A**) ∣0*_L_*〉 state of the Bacon-Shor code is encoded into nine physical qubits by preparing three copies of a three-qubit GHZ-entangled state. (**B**) Shor-style checks are performed by mapping syndromes *S*_1_ = *Z*_1_*Z*_2_*Z*_3_*Z*_4_*Z*_5_*Z*_6_ and *S*_2_ = *Z*_4_*Z*_5_*Z*_6_*Z*_7_*Z*_8_*Z*_9_ to qubits 10 and 11 serving as ancillae. Separate ancilla qubits (12 and 13) are used to repeat the measurement. (**C**) Steane-style syndrome extraction involves preparing a ∣+*_L_*〉 logical state using the circuit on the left applied to qubits 10 to 18 and then performing a transversal CNOT between the data qubit and the ancilla qubit. (**D**) Ion positions in the 23-qubit ion used for Shor-style syndrome extraction. (**E**) Ion positions in the 23 ion chain used for Steane-style syndrome extraction.

In this work, we experimentally compare Shor and Steane methods applied to the [[9,1,3]] subsystem Bacon-Shor code (*24*). The stabilizer group of this code is generated by four weight-6 operators: *S*_1_ = *Z*_1_*Z*_2_*Z*_3_*Z*_4_*Z*_5_*Z*_6_, *S*_2_ = *Z*_4_*Z*_5_*Z*_6_*Z*_7_*Z*_8_*Z*_9_, *S*_3_ = *X*_1_*X*_2_*X*_4_*X*_5_*X*_7_*X*_8_, and *S*_4_ = *X*_2_*X*_3_*X*_5_*X*_6_*X*_8_*X*_9_. The values of 12 weight-2 symmetry operators *Z_i_Z*_*i*+3_ (*i* ∈ {1,2,3,4,5,6}) and *X_i_X*_*i*+1_ (*i* ∈ {1,2,4,5,7,8}), referred to as gauge operators, can freely fluctuate without destroying the logical information. The logical ∣0*_L_*〉 (∣+*_L_*〉) state consists of three rows (columns) of physical $\frac{1}{\sqrt{2}}\left(|+++\rangle +|---\rangle \right)$ $\left[\frac{1}{\sqrt{2}}(|000\rangle +|111\rangle )\right]$ Greenberger-Horne-Zeilinger (GHZ) states for a particular gauge and can be prepared fault-tolerantly with only unitary operations (*18*). We prepare the ∣0*_L_*〉 logical state using the circuit shown in Fig. 1A. In this circuit, any single- or two-qubit error of weight-1 is equivalent to a single error together with the action of a gauge operator. The logical operator *Z_L_* = *Z*_1_*Z*_2_*Z*_3_ can be fault tolerantly measured by transversal *Z* readouts followed by a majority vote among the row parities *Z*_3*i*+1_*Z*_3*i*+2_*Z*_3*i*+3_ (*i* = 0,1,2).

The canonical method to determine the error syndrome of Bacon-Shor code is to measure weight-2 gauges (*24*–*26*). The values of four stabilizer generators can be obtained by products of gauge measurement outcomes. An alternative approach is to measure the four stabilizer generators directly with single qubit ancilla (*27*). This method depends on a specific ordering of the circuit that follows the underlying gauges (*28*). The circuit for measuring the *Z* stabilizers *S*_1_ and *S*_2_ is presented in Fig. 1B. From a broad perspective, both of these methods fall under the category of Shor EC. Measuring the full-syndrome requires 24 to 48 two qubit gates between the data qubits and syndrome qubits in an adaptive approach (*18*).

In Steane EC (*20*), a transversal controlled-not (CNOT) gate is applied between data and a fault tolerantly prepared ∣+*_L_*〉 (∣0*_L_*〉) ancilla resource state used for correcting *X* (*Z*) errors, as illustrated in Fig. 1C. When correcting *X* (*Z*) errors, the data (ancilla) block is the control of CNOT, while the ancilla (data) block is the target. After the transversal CNOT gate, all qubits are measured in *Z* (*X*) basis. The error syndrome is obtained by parities of *Z* (*X*) stabilizer elements. We then assume that the syndrome is perfect and apply correction immediately based on the syndrome. Steane EC requires only 18 two qubit gates for the Bacon-Shor code to touch the data or teleport the data, respectively. For any code, when gate errors dominate, it is expected that Steane or Knill EC will outperform Shor EC. The cost is the preparation of the complicated ancilla qubits. For the case of the distance three Bacon-Shor code, the logical state can be directly prepared fault tolerantly using unitary preparation (*3*). This is a key advantage for small circuits and greatly benefits a Steane EC circuit.

## RESULTS

We use an ion trap quantum computer to test the hypothesis that Steane EC induces less noise on the logical qubits than Shor EC. To perform this test, we prepare the logical ∣0〉 state of the Bacon-Shor code (∣0*_L_*〉) and check the value of its logical *Z* operator using an error-corrected measurement after a single EC cycle. We also limit our interrogation to measuring the *X* error syndrome. A full EC cycle would require measuring both the *X* and the *Z* error syndromes.

The experimental system, based on (*3*), consists of a near-equispaced linear chain of ^171^Yb^+^ ions storing ground-state hyperfine qubits. The ions are held in a Sandia HOA-2.1.1 trap housed in a room-temperature ultrahigh vacuum chamber and are manipulated using a 355-nm Raman process driven by single global laser beam together with up to 32 individually addressed beams. The state of each qubit is read out using an array of photomultiplier tubes coupled to multimode fibers.

To allow a direct comparison between fault-tolerant Shor and Steane EC, we extended our experimental system from 15 to 23 ions. The increase in the number of ions leads to a sharp increase in their axial motion and the attendant errors in their individual addressing (*29*). To counter these errors, we confined the ions to decrease the chain spacing from 4.4 to 3.8 μm and tailored our gates to suppress coupling to the chain’s axial motion, as described in Materials and Methods. The mapping from qubits to ions for both Shor and Steane EC experiments are shown in Fig. 1 (D and E, respectively). The CNOT and Hadamard gates are decomposed in a standard way into native Mølmer-Sørensen gates and single-qubit rotations (*30*). Details of the physical circuit model can be found in the Supplementary Materials.

To demonstrate our ability to control errors in our larger system, we perform a logical state preparation and measurement (SPAM) test. After preparing the ∣0*_L_*〉 state, we measure all qubits in the *Z* basis and decode the logical *Z*-measurement outcome via majority voting. The logical SPAM error rate, defined as the probability of measuring *Z_L_* = −1, is observed to be $0.20_{-0.14}^{+0.27}\%$, which is comparable with the previous result of 0.23(0.13)% in (*3*).

### Shor EC

Our baseline experiment is a Shor-style syndrome extraction of the two *Z* stabilizers following the previous work (*3*). The Shor EC procedure for *X* errors involves measuring two weight-6 *Z* stabilizer generators *S*_1_ and *S*_2_ separately.

A fault-tolerant Shor EC cycle requires measuring the checks in multiple rounds. An adaptive protocol for general distance three quantum error-correction code (*27*, *31*) is to measure the stabilizer generators once and repeat the measurement if a nonzero error syndrome is detected. (*31*).

We use our 23 ion system to perform up to two rounds of *Z*-syndrome extraction by mapping *Z* stabilizers to four ancilla qubits as shown in Fig. 1B. We denote the *Z* syndrome obtained in round *r* = 1,2 by a two-bit string $s^{\left(r\right)}=s_{1}^{\left(r\right)}s_{2}^{\left(r\right)}$, where $s_{i}^{\left(r\right)}=1$ (0) if the measurement of *S_i_* in round *r* outputs −1 (*1*). We decode the experimental data using three different decoders:

1) Single-shot decoder: We perform only a single extraction round and immediately apply correction based on *s*^(1)^. The correction operators corresponding to *s*^(1)^ = 00, 01, 11, and 10 are *I*, *X*_1_, *X*_4_, and *X*_7_, respectively. This decoder is not fault-tolerant because a single *X* error between the two CNOT gates on qubits 4, 5, or 6 will always result in syndrome *s*^(1)^ = 01. After correction, an additional *X* error will be introduced on qubit *7*; the original error is amplified and becomes uncorrectable.

2) Adaptive decoder I: If *s*^(1)^ = 00, then we do not perform the next extraction round and no correction is applied; otherwise, the next extraction round is triggered. As one error already occurred in round 1, we assume that *s*^(2)^ is trustworthy and apply correction based on *s*^(2)^ only. The correction operators corresponding to *s*^(2)^ = 00, 01, 11, and 10 are *I*, *X*_1_, *X*_2_, and *X*_3_, respectively.

3) Adaptive decoder II: The adaptive decoder I can be improved by noticing that the second extraction round is unnecessary when *s*^(1)^ is equal to 10 or 11. The weight-1 faults compatible with *s*^(1)^ = 10 are a single-qubit *X* error on qubits 1 to 3 before the two-qubit gates, or a single measurement error on $s_{1}^{\left(1\right)}$. We apply the correction *X*_1_ immediately to correct the data-qubit error (up to a gauge) or convert the measurement error into a data-qubit error without amplification of errors. The only weight-1 faults compatible with *s*^(1)^ = 11 are single-qubit *X* errors on qubits 4, 5, or 6 before all two-qubit gates, which are corrected by *X*_2_. If *s*^(1)^ = 01, then another extraction round is performed and the correction is applied based on *s*^(2)^.

**Fig. 2.**
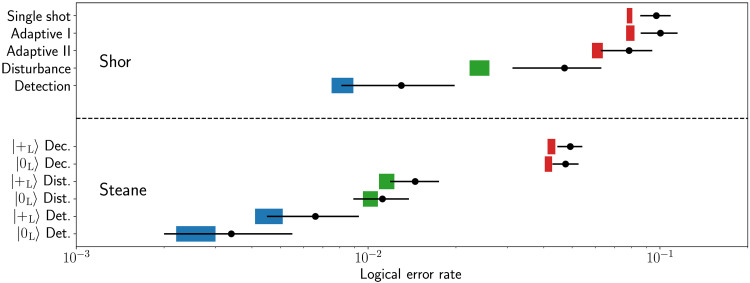
Comparison of Shor-style and Steane-style EC protocols with the ancilla in the ∣0*_L_*〉 and ∣+*_L_*〉 logical states. Decoder (Dec., red) corrects the logical qubit with syndrome information before the final correction based on logical qubit measurement. Disturbance (Dist., green) only corrects based on the logical qubit measurement. Detection (Det., blue) only keeps the data when no errors are detected on the ancilla qubits. The Shor disturbance measurement reflects only a single round of syndrome measurement. The black dots are the experimental data, and the bars are numerical simulations. The errors correspond to 95% confidence intervals. The simulations are based on error characterization experiments and are not adjusted for the logical qubit experiments.

Our current experimental setup does not support mid-circuit measurements and therefore cannot execute adaptive circuits. To estimate the logical error rate of adaptive decoders, we post-select data from two experiments, denoted by *E*_1_ and *E*_2_, in which either one or two extraction rounds are deterministically performed. Let μ[*s*^(1)^] be the probability that the first-round syndrome *s*^(1)^ is measured, and let λ*_r_*[*s*^(1)^] (*r* = 1,2) be the conditional logical error rate in experiment *E_r_* with first-round syndrome *s*^(1)^ when *s*^(*r*)^ is used for correction. The total logical error rates for single-shot decoder, adaptive decoder I, and adaptive decoder II are calculated as

$$\begin{array}{c} p_{L}^{\left(\text{SS}\right)}=\mu \left(00\right)\lambda_{1}\left(00\right)+\mu \left(10\right)\lambda_{1}\left(10\right)+\mu \left(11\right)\lambda_{1}\left(11\right)+\mu \left(01\right)\lambda_{1}\left(01\right),\\ p_{L}^{\left(A1\right)}=\mu \left(00\right)\lambda_{1}\left(00\right)+\mu \left(10\right)\lambda_{2}\left(10\right)+\mu \left(11\right)\lambda_{2}\left(11\right)+\mu \left(01\right)\lambda_{2}\left(01\right),\\ p_{L}^{\left(A2\right)}=\mu \left(00\right)\lambda_{1}\left(00\right)+\mu \left(10\right)\lambda_{1}\left(10\right)+\mu \left(11\right)\lambda_{1}\left(11\right)+\mu \left(01\right)\lambda_{2}\left(01\right) \end{array}$$ (1)

respectively. We note that the values μ[*s*^(1)^] for *E*_1_ and *E*_2_ agree with negligible difference. In our experiment, we find that $p_{L}^{\left(\text{SS}\right)}=9.7\left(1.2\right)\%$ (first row in Fig. 2), $p_{L}^{\left(A1\right)}=10.0\left(1.5\right)\%$ (second row in Fig. 2), and $p_{L}^{\left(A2\right)}=7.8\left(1.6\right)\%$ (third row in Fig. 2). We see that adaptive decoder II already outperforms single-shot decoder in the experiment. The experimental values of λ*_r_*, which are included in the Supplementary Materials, show that λ_1_[*s*^(1)^] < λ_2_[*s*^(1)^] for *s*^(1)^ = 00,10,11, while λ_1_(01) > λ_2_(01). This verifies that repetitive syndrome extraction helps when *s*^(1)^ = 01, for which single-shot decoder fails to maintain fault tolerance.

We also consider the use of one-round Shor-style circuit for post-selection of ∣0*_L_*〉, whose logical error and rejection rates are λ_1_(00) and 1 − μ(00). From Table 1, we see that Shor-style post-selection introduces much more logical *X* errors than direct preparation of ∣0*_L_*〉 ($1.30_{-0.49}^{+0.68}\%$ versus $0.20_{-0.14}^{+0.27}\%$), with the extra cost of nonvanishing rejection rate.

**Table 1.** Comparison of post-selection protocols for between Shor- and Steane-style error detection circuits. ∣0*_L_*〉 We calculate the logical error rate (LER) based on the experimental (EXP) data, our simulation (SIM) of the experiment, and our predicted simulation for future experimental improvements (IMP). Post-selection leads to a rejection rate (RR), which is the fraction of trials that are not post-selected.

Postselection protocol		Direct ∣0*_L_*〉 preparation	Shor	Steane (∣+*_L_*〉 ancilla)	Steane (∣0*_L_*〉 ancilla)
LER	EXP	$0.20_{-0.14}^{+0.27}\%$	$1.30_{-0.49}^{+0.68}\%$	$0.66_{-0.21}^{+0.27}\%$	$0.34_{-0.14}^{+0.21}\%$
	SIM	$0.22_{-0.03}^{+0.03}\%$	$0.82_{-0.07}^{+0.07}\%$	$0.46_{-0.05}^{+0.05}\%$	$0.26_{-0.04}^{+0.04}\%$
	IMP	$0.14_{-0.02}^{+0.03}\%$	$0.42_{-0.04}^{+0.05}\%$	$0.06_{-0.02}^{+0.02}\%$	$0.06_{-0.01}^{+0.02}\%$
RR	EXP		(35.32 ± 1.87)%	(27.10 ± 1.44)%	(33.81 ± 1.32)%
	SIM		(33.04 ± 0.29)%	(33.67 ± 0.36)%	(32.16 ± 0.35)%
	IMP		(20.71 ± 0.25)%	(15.98 ± 0.25)%	(12.32 ± 0.23)%

### Steane EC

Our Steane EC experiment involves the prepartion of the data qubits in the ∣0*_L_*〉 state and the syndrome qubits in the ∣+*_L_*〉 state. A transversal CNOT between the data qubits and the syndrome qubits allows *X* errors in the preparation of the data qubits to be mapped to the syndrome qubits (1c). For arbitrary data states, the logical ancilla state should be prepared in ∣+*_L_*〉, which is not affected by the logical CNOT for any ideal input logical state. For the ∣0*_L_*〉 data state, we can also use another ∣0*_L_*〉 state as ancilla. We test both ancilla states and find a logical error on the data qubit of 4.93(0.49)% for a ∣+*_L_*〉 ancilla (row 6 in Fig. 2) and a 4.75(0.5)% a ∣0*_L_*〉 (row 7 in Fig. 2) after we correct based on the ancilla qubit measurements. We find that the Steane method yields lower logical data error compared to the Shor syndrome readout with *P* value of at least 3.7%.

Another way to measure the negative impact of the syndrome measurement circuit on the code is to decode the final logical measurement without the use of any syndrome information from the intermediate EC cycle. The corresponding logical error rate is termed as disturbance. For both Shor and Steane EC, we consider the disturbance of a single syndrome extraction round. We find that the resulting disturbance of the data qubits (Fig. 2) is at least 70(12)% less for Steane EC than for Shor EC .

When we use a Steane-style circuit with post-selection, our state will be in the wrong logical state with probability *O*(*p*^3^) for a probabilistic error model. Our state post-selected on errors measured when the ancilla qubit is prepared in the ∣+*_L_*〉 state is $0.66_{-0.21}^{+0.27}\%$ (row 10 in Fig. 2), which outperforms the Shor-based post-selection (row 5 in Fig. 2). If we use an ancilla in the ∣0*_L_*〉 state, then we can also post-select on logical errors. This further improves the logical fidelity output to $0.34_{-0.14}^{+0.21}\%$ (last row in Fig. 2), which is an improvement over not checking the error syndrome at the cost of a 34% rejection rate.

### Simulation and perspective

We developed a simulation of the experiment based on unitary evolution of the full wave function where errors are added probabilistically as unwanted unitary transformations. The applied unitaries are derived from experimentally measured errors in SPAM, single qubit gates, two-qubit gates including cross-talk errors, and the effects of axial heating of the ion chain. The full details are in the Supplementary Materials.

The simulation is not fit to the logical error data and the parameters are instead determined from independent characterization and calibration experiments. This results in non-identical errors on different qubits in the chain. We see broad agreement between the simulated and the experimental data across the measured parameters.

The simulation allows us to determine the best steps forward for reducing the logical error. We find that the dominant source of error is the heating of the lowest axial mode at the rate of 180 phonons/ms, which causes noise on the Rabi frequencies on all ions (*29*). The single- and two-qubit gates in the EC circuit experience random under-rotation errors that grow with time. Our simulation method considers the time correlations of under-rotation errors within the circuit. While small single-qubit under-rotation errors are reduced by SK1 composite pulse sequence (*32*), the XX gates are strongly affected. Another significant source of error is the dephasing during two-qubit gates. In our experiments, the effect of cross-talk on logical gate performance is negligible.

The axial heating can be reduced by cooling the trap (*33*), preparing the trap surface (*34*), or both (*35*), and its effects can be countered by sympathetic cooling (*29*). The reduction of axial temperature together with a decrease in the axial phase gradients of the addressing beams should also reduce the two-qubit dephasing (*36*). Dephasing can be further reduced by suppressing the relative intensity fluctuations of the red and blue sidebands in a (fiber-coupled) double-pass acousto-optic modulator (AOM) setup.

A limitation in our experiment is that detecting error syndromes does not help the decoding of the logical qubit without post-selection. In all cases, it would be better not to apply the syndrome extraction gadget. Using the simulator, we explore a scenario where the heating rate is reduced to 0, and *Z* (*X*) errors during two-qubit gates is reduced by a factor of 5 (*4*). We assume that the initial temperature of the lowest axial mode remains unchanged at 5.5 mK.

In this limit, we find that the logical error rate decreases to 0.81(0.06)% for Steane EC and 2.45(0.16)% for Shor EC (Table 2). Both methods see substantial improvements in errors, and Steane EC continues to outperform the Shor EC. The post-selected states would be prepared with a lower rejection rate. For Steane with ∣0*_L_*〉 ancilla, we see a reduction of the rejection rates by a factor of 3 and of the error rates by a factor of 4 over the current simulated values (Table 1).

**Table 2.** Comparison between Shor and Steane EC protocols. We calculate the LER and disturbance (DSTB) based on the EXP data, our SIM of the experiment, and our predicted simulation for future experimental IMP. DSTB is the logical error rate when no ancilla readout information is used. Details of the protocols are in the Results section of the main text.

EC protocol		Shor			Steane	
		Single shot	Adaptive I	Adaptive II	∣+*_L_*〉	∣0*_L_*〉
LER	EXP	(9.72 ± 1.16)%	(10.04 ± 1.45)%	(7.84 ± 1.57)%	$4.93_{-0.48}^{+0.51}\%$	$4.75_{-0.47}^{+0.51}\%$
	SIM	(7.87 ± 0.17)%	(7.92 ± 0.26)%	(6.11 ± 0.26)%	(4.25 ± 0.13)%	(4.15 ± 0.12)%
	IMP	(5.68 ± 0.14)%	(3.11 ± 0.17)%	(2.45 ± 0.16)%	(0.81 ± 0.06)%	(1.08 ± 0.07)%
DSTB	EXP	(4.71 ± 1.59)%			$1.45_{-0.26}^{+0.30}\%$	$1.12_{-0.23}^{+0.26}\%$
	SIM	(2.41 ± 0.10)%			(1.16 ± 0.07)%	(1.02 ± 0.06)%
	IMP	(1.49 ± 0.08)%			(0.18 ± 0.03)%	(0.21 ± 0.03)%

## DISCUSSION

Our work has demonstrated the promise of Steane-style quantum EC. The next level of investigation requires intermediate measurements to measure and reset the Steane ancilla. A larger system capable of pairing up multiple ion chains would allow us to perform Knill-style syndrome extraction via teleportation. Knill’s method is both efficient and prevents the unwanted accumulation of leakage errors to states outside of the qubit space. Leakage errors present a challenge to Shor and Steane-style EC without additional leakage reduction gadgets (*37*). Further work should focus on examining codes with larger distance where the challenge of preparing logical ancilla state increases, but the disturbance to data introduced by Steane/Knill EC circuit also decreases. It would also be interesting to investigate intermediate error-correction circuits in between Shor and Steane methods (*22*).

The procedure outlined here is ideal for quantum architectures that enable low-error rate shuttling to connect distant qubits without multiple swap operations. For ions, a charge-coupled device–type architecture (*38*) that uses longer than two-ion chains as a baseline would work well. Ion temperature can be lowered after merging chains using sympathetic cooling and benefits from precooling one of the two merged chains. For neutral atoms, a change in encoding procedures from the Steane code (*13*) would enable testing these results in that system. In future quantum dot systems, which allow coherent transport of spins, a similar result will hold (*39*). Steane EC is also promising for a multilayer architecture where a logical ancilla qubit layer can be directly coupled to the data qubit layer.

## MATERIALS AND METHODS

### Experimental system

**Fig. 3.**
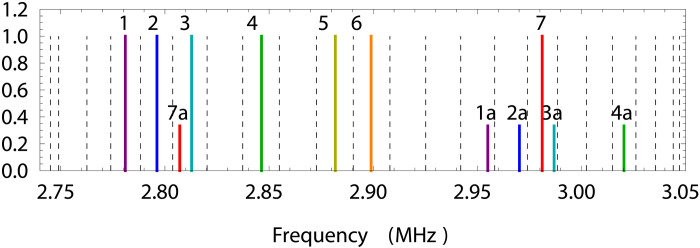
Choice of carrier frequencies for amplitude-modulated XX gates in long ion chain. The frequencies of the radial modes of 23 ions (dashed lines) compared to the mean frequencies of the state-dependent amplitude-modulated forces (lines 1 to 7) that are used to perform entangling gates. Because of motion of the lowest axial mode of the ion chain, the gate waveforms as seen by the ions acquire sidebands at ±174 kHz (lines 1a to 7a).

To perform two-qubit Mø lmer-Sø rensen gates, we use individually addressed Raman beams to induce state-dependent amplitude-modulated forces that couple the internal states of the ions to one set of the ions’ radial modes (Fig. 3). For robustness to mode frequency drift, we choose the mean frequencies of these forces (lines 1 to 7 on Fig. 3) half-way between the frequencies of the neighboring radial modes (dashed lines on Fig. 3) and use a time-reversal symmetric modulation waveforms that null the mean motional displacement of each mode during the gate (*40*).

Predominant axial motion of the ions at the lowest axial mode frequency of 174 kHz causes the gate waveforms experienced by the ions to acquire modulation sidebands (lines 1a to 7a in Fig. 3). To suppress the resulting errors, we choose the XX gate waveforms for which these sidebands are separated from the radial modes by at least 3.3 kHz.

To mitigate the impact of the axial ion motion on the single-qubit gates, we first align the addressing beams to minimize the phase gradient of the Raman coupling along the chain axis.

The effects of Raman amplitude fluctuations are countered by using SK1 pulses (*32*). Each 13-μs-long pulse segment of the compound pulse is further Gaussian-shaped so as to null the first-order contribution of the ions’ motion at the lowest axial frequency to the resulting unitary.

### Physical error rates and error modeling

The logical error rate for each experiment is numerically estimated via Monte-Carlo sampling of state vector evolutions. The leading source of errors in the experiment is the motion of the ion chain in the lowest axial mode (mode 0) with frequency ω_0_ = 2π × 174 kHz, which misaligns the ions relative to the individual-addressing beams. These beams are nearly Gaussian-shaped with waist *w* = 450 nm, corresponding to instantaneous carrier Rabi frequency of *i*-th ion $\Omega_{i}=\Omega_{i,\text{max}}\text{exp}\left(-x_{i}^{2}/w^{2}\right)$, where *x_i_* is the instantaneous position of this ion.

Because, in our experiments, the energy *E*_0_ of mode 0 corresponds to hundreds of motional quanta ℏω_0_, we describe this motion classically. In this limit, Ω*_i_* varies with the classical phase ϕ of the ions’ motion as Ω*_i_* = Ω_*i*, max_ exp (−2ϵ*_i_*sin^2^ϕ), where $\epsilon_{i}=b_{i0}^{2}\hbar {\mathrm{nE}}_{0}/{\mathrm{mw}}^{2}\omega_{0}^{2}$ is a unitless decay parameter, *m* is the ion mass, and *b*_*i*0_ is the participation of ion *i* in mode 0. Averaging over the motional phase ϕ gives the mean Rabi frequency ${\bar{\Omega }}_{i}=\Omega_{i,\text{max}}\;f\left(\epsilon_{i}\right)$ of ion *i*, where *f*(*x*) = *e*^−*x*^*I*_0_(*x*), with *I*_0_ as the modified Bessel function of the first kind.

Uncertainty in the energy *E*_0_ introduces rotation errors in both single- and two-qubit gates (*29*). A carrier rotation of ion *i* by angle θ becomes an imperfect rotation by angle θ*f*(ϵ*_i_*), while a two-qubit *XX*(θ) between ions *i* and *j* gate becomes *XX*[θ*f*(ϵ*_i_*)*f*(ϵ*_j_*)]. Note that, in our model, ϵ*_i_* and ϵ*_j_* are perfectly correlated because they are both derived from the same random number *n* of motional quanta as ϵ_*i*,*j*_ = *nu*_*i*,*j*_, with $u_{i,j}=b_{\left(i,j\right)0}^{2}\hbar /\left({\mathrm{mw}}^{2}\omega_{0}\right)$ fixed unitless numbers that we calculate.

**Fig. 4.**
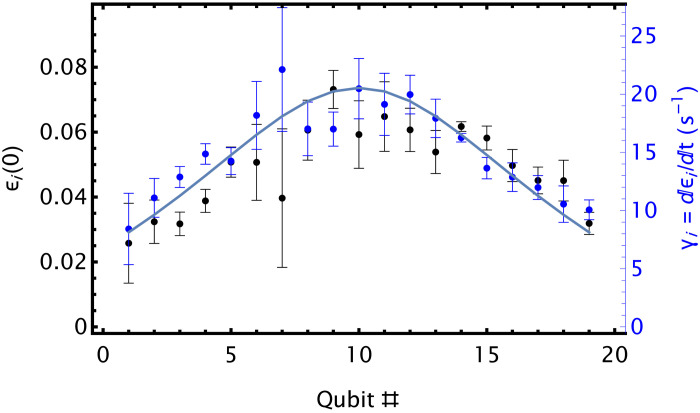
Effect of axial heating on ion addressing. The decay parameter (*29*) ϵ*_i_* of carrier Rabi oscillations as function of qubit number at the beginning of the circuit (black) and the rate of its increase with time γ*_i_* (blue). Fits of the data to $\epsilon_{i},\gamma_{i}∼b_{i0}^{2}$ (solid line) yield the initial mean phonon number in the lowest axial mode $\bar{n}=660\left(20\right)$ and the heating rate of the lowest axial mode of the chain of $\overset{\cdot }{\bar{n}}=$180(4) phonons/ms.

We assume that at beginning of the circuit (*t* = 0) mode 0 is in thermal equilibrium. In this case, ϵ*_i_* will be Boltzmann-distributed with mean ${\bar{\epsilon }}_{i}\left(0\right)$, which we determine by fitting carrier Rabi oscillations of all the qubits to the model from (*29*) (Fig. 4). We fit ${\bar{\epsilon }}_{i}$ to $\bar{n}u_{i}$ to obtain the mean initial phonon number $\bar{n}\left(0\right)=660\left(20\right)$. At the beginning of the simulation, we sample *n* from from the Boltzmann distribution with mean $\bar{n}\left(0\right)$.

Without mid-circuit sympathetic cooling, the energy of the lowest axial mode increases with time, causing the gates applied near the end of the circuit to perform worse. We determine the rate of increase γ*_i_* of ϵ*_i_* by fitting Rabi oscillations at later times in the circuit (Fig. 4). A fit of γ*_i_* to $\overset{\cdot }{\bar{n}}\times u_{i}$ yields the axial heating rate $\overset{\cdot }{n}=$ 180 (*4*) phonons/ms. This is consistent with a noisy electric field that is homogenous across the chain and corresponds to the heating rate of 8.3(2) phonons/ms of a single ion with axial frequency of 2π× 174 kHz.

We model heating of mode 0 as a biased random walk in *n*. Given *n*(*r*Δ*t*), we set

$$n\left[\left(r+1\right)\Delta t\right]≔\left\{\begin{array}{cc} n\left(r\Delta t\right)+1 & \text{withprob}.\;\left(n+1\right)\times \overset{\cdot }{\bar{n}}\Delta t\\ n\left(r\Delta t\right)-1 & \text{withprob}.\;n\times \overset{\cdot }{\bar{n}}\Delta t\\ n\left(r\Delta t\right) & \text{otherwise} \end{array}\right.$$ (2)

To determine the imperfect rotation angle for a gate starting at time *t*, for each raw single-qubit rotation σ_ϕ_(θ) in the *x*-*y* plane on ion *i*, the actual rotation angle $\tilde{\theta }$ is set to be

$$\tilde{\theta }≔\theta \left[1+u_{i}\bar{n}\left(0\right)\right]f\left[u_{i}n\left(t\right)\right]$$ (3)

The extra coefficient $\left[1+u_{i}\bar{n}\left(0\right)\right]$ compensates the average under-rotation at *t* = 0. Similarly, for each two-qubit *XX*(θ) rotation between ions *i* and *j* starting at time *t*, the actual rotation angle $\tilde{\theta }$ is set to be

$$\tilde{\theta }≔\theta \left[1+u_{i}\bar{n}\left(0\right)\right]\left[1+u_{j}\bar{n}\left(0\right)\right]f\left[u_{i}n\left(t\right)\right]f\left[u_{j}n\left(t\right)\right]$$ (4)

We ignore the fluctuation of *n*(*t*) within a single- or two-qubit gate duration.

**Fig. 5.**
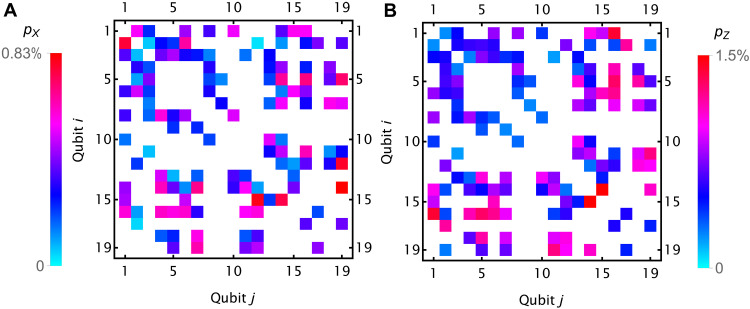
X-flip errors in XX gates. The measured probabilities *p_X_* of an *X* error (**A**) and *p_Z_* of a *Z* error (**B**), per qubit, per fully entangling *XX* gate between qubits *i* and *j*.

Another contributing factor to logical error is dephasing during the entangling gates. Sources of this dephasing include axial phase gradients in the individual addressing beams combined with axial motion of the ions, fluctuations, in the ratio of the red and blue sidebands and fluctuations in coupling to other Zeeman levels. We model the dephasing during the XX gate between qubits *i* and *j* as a pair of equal single-qubit phase-flip Pauli channels

$$E_{Z}\left(\rho \right)=\left[1-p_{Z}^{\left(i,j\right)}\right]\rho +p_{Z}^{\left(i,j\right)}Z\rho Z$$ (5)

on qubits *i* and *j* following the gate. We determine the error probability $p_{Z}^{\left(i,j\right)}$ for each ion pair by performing *K* paired maximally entangling gates with opposite rotation angles (+*XX*/−*XX*) inside a Ramsey sequence on both ions. We fit the resulting Ramsey fringes to extract the contrast for both ions and set $p_{Z}^{\left(i,j\right)}$ to 1/2 of the average measured contrast loss per gate (Fig. 5). We assume $p_{Z}^{\left(i,j\right)}$ to be time independent.

To model dephasing on idling qubits due to optical phase fluctuations and unwanted ac shifts, for each idling circuit location with duration *t*, we apply a Pauli *Z* error with probability Γ_deph_*t*, where Γ_deph_ is the measured dephasing rate. For each measurement, we flip the outcome from ∣1〉 (∣0〉) to ∣0〉 (∣1〉) with probability *p*_1→0_ = 4 × 10^−3^ (*p*_0→1_ = 1.5 × 10^−3^).

**Fig. 6.**
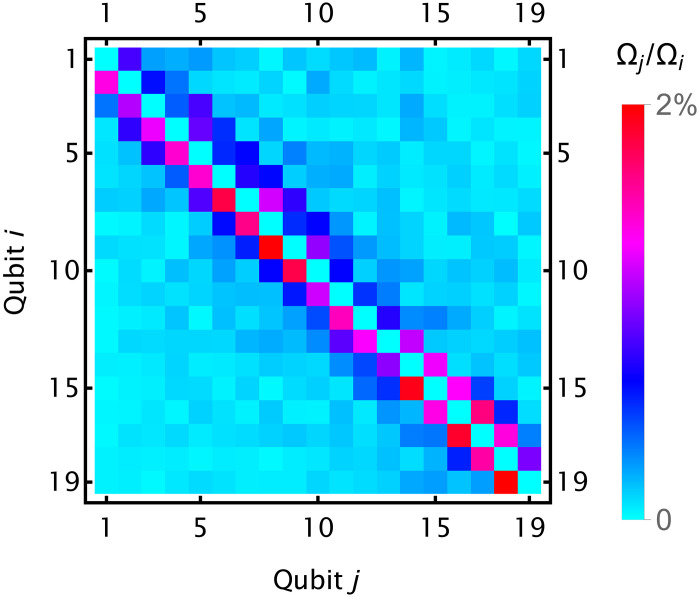
Ion addressing cross-talk. The measured ratio of the Rabi frequency Ω*_j_* of qubit *j* and the Rabi frequency Ω*_i_* of the target resonantly driven qubit *i*.

Acoustic and electrical cross-talk in the 32-channel AOM leads to cross-talk in individual addressing. We characterize this cross-talk by measuring the ratio of the carrier Rabi frequencies for a single addressed ion and the remaining qubits (Fig. 6). The effects of cross-talk on single-qubit gates are suppressed using SK1 pulses.

We estimate the contribution of two-qubit cross-talk errors on logical infidelity. When a two-qubit gate *X_i_X_j_*(θ) is applied between ions *i* and *j* with carrier Rabi frequency Ω(*t*), each other ion *k* ≠ *i*, *j* will also experience a spin-dependent force with carrier Rabi frequency Ω*_k_*(*t*) = (χ_*i*,*k*_ + χ_*j*,*k*_)Ω(*t*), where χ_*i*,*k*_ are the carrier cross-talk ratios. After the two-qubit drive is finished, two small two-qubit rotations $X_{i}X_{k}\left(\theta_{k}^{\left(1\right)}\right)$ and $X_{j}X_{k}\left(\theta_{k}^{\left(2\right)}\right)$ will be applied between ion pairs (*i*, *k*) and (*j*, *k*). Let *A*_*i*,*j*,*k*_ be the rotation angle on ion pair (*i*, *k*) when the two-qubit AM pulse sequence on the pair (*i*, *j*) is applied on the pair (*i*, *k*) instead. Then, we have

$$\theta_{k}^{\left(1\right)}=\left(\chi_{i,k}+\chi_{j,k}\right)A_{i,j,k}$$ (6)

and

$$\theta_{k}^{\left(2\right)}=\left(\chi_{i,k}+\chi_{j,k}\right)A_{j,i,k}$$ (7)

The values *A*_*i*,*j*,*k*_ can be analytically calculated from the pulse shapes, motional mode frequencies, and spin-motion coupling strengths.

Motional dephasing during two-qubit gates causes *X* errors (*41*) and contributes to the logical error rate. We determine the probability *p_X_* of single-qubit Pauli *X* errors during the *X_i_X_j_*(θ) gate between ions *i* and *j* by preparing the target qubits in the ∣0〉 state and measuring the probability *p* of a single bit flip after *K* fully entangling gates. We set *p_X_* = *p*/*K*/2 and show the results in Fig. 5B. See “Demonstration of fault-tolerant Steane quantum error correction” by the research groups led by M. Müller (Aachen & Jülich) and by T. Monz (Innsbruck) for related work (*42*).
