# Supplementary material for: Comparing Shor and Steane error correction using the Bacon-Shor code
Source: Sci Adv. 2024 Nov 6;10(45):eadp2008. doi: 10.1126/sciadv.adp2008 (PMC11800988; doi:10.1126/sciadv.adp2008)
Supplement: Supplementary file 1 — Supplementary Text Figs. S1 to S3 Tables S1 to S3 [file sciadv.adp2008_sm.pdf]

Science Advances
AAAS

Supplementary Materials for

Comparing Shor and Steane error correction using the Bacon-Shor code

Shilin Huang *et al.*

Corresponding author: Marko Cetina, marko.cetina@duke.edu

Sci. Adv. **10**, eadp2008 (2024)
DOI: 10.1126/sciadv.adp2008

This PDF file includes:

Supplementary Text
Figs. S1 to S3
Tables S1 to S3

Fault-Tolerant Protocols Here we describe the fault-tolerant protocols implemented in our experiments, which include circuits for logical state preparation and measurement (SPAM) and syndrome extraction, decoders and calculation of logical error rates (LER). We focus on how syndrome extraction circuits disturb the logical $|0_L\rangle$ state of the $[[9, 1, 3]]$ Bacon-Shor code and only consider logical bit-flip (X_L) errors when calculating LER. We compare experiment results with numerical simulations to benchmark our error modeling, and provide predictions of LER assuming improved experimental parameters. In our prediction, we assume that sympathetic cooling will cool down the lowest axial mode to initial temperature before each single- and two-qubit gate is performed, which resets the variable ϵ by a random number from the Boltzmann distribution. We also assume a $5\times$ reduction of Z errors during the two-qubit gates and a $4\times$ reduction of X errors from imperfect spin-motion disentanglement. For all the result tables, we use LER and RR to denote logical error rate and rejection rate of postselection, and EXP, SIM, IMP to denote rates extracted from experimental data, simulations with measured experimental parameters during the preparation of this work and simulation with anticipated improvement of experimental parameters.

Logical State Preparation and Measurement The implementations for $|0_L\rangle$ and $|+_L\rangle$ state preparations using trapped-ion native operations are shown in Figure S1. In these circuits, any single- or two-qubit error of weight 1 is equivalent to a single error on the code block up to gauges.

Shor-Style Syndrome Extraction The measurement circuit for each syndrome extraction round using trapped-ion native gates is shown in Figure S2, which only utilizes two ancilla qubits. Our choice of two-qubit gate scheduling guarantees that any single fault an ancilla only propagate one error to data up to gauges.

Table S1 shows the values of $\mu_r(s^{(1)})$ and $p_r(s^{(1)})$ in both experiments and simulations

together with the values of the *conditional disturbance* $\delta_r(s^{(1)})$, defined as the logical error rate of E_r conditioned on $s^{(1)}$ without the use of ancilla information $s^{(i)}$. In most cases $\lambda_r(s^{(1)}) > \delta_r(s^{(1)})$, indicating that ancilla information is not helping. We found that simulation values of δ_r are all about 40% smaller than experiment values. In our circuit, the majority of disturbance is contributed from dephasing errors in two-qubit gates. This mismatch might indicate that the dephasing errors in two-qubit gates can be time-dependent and correlated.

The experimental and theoretical values of logical error rates are presented in Table 2. We see that adaptive decoder II already outperforms single-shot decoder in the experiment. Simulation shows that adaptive decoder I will also outperforms single-shot with improved error parameters, which further verifies that repetitive stabilizer measurements are necessary in Shor-style error correction.

Steane-Style Syndrome Extraction In Steane error correction,a transversal CNOT gate is applied between data and a fault-tolerantly prepared $|+_L\rangle$ ($|0_L\rangle$) ancilla resource state used for correcting X (Z) errors. When correcting X (Z) errors, the data (ancilla) block is the control of CNOT, while the ancilla (data) block is the target. After the transversal CNOT gate, all qubits are measured in Z- (X-) basis. The error syndrome is obtained by parities of Z- (X-) stabilizer elements. We then assume the syndrome is perfect and apply correction immediately based on the syndrome. In our Steane EC experiment, we first sequentially prepare a $|0_L\rangle$ state for data and a $|+_L\rangle$ state for ancilla, then apply transversal CNOT between data and ancilla, and finally measure all physical qubits in Z basis. The physical implementation of each CNOT using trapped-ion operations is presented in Figure S3. The CNOT gates are applied sequentially in our experiment.

For benchmarking purposes, we also consider replacing $|+_L\rangle$ by $|0_L\rangle$ for X error correction. Normally this does not work as it performs a logical Z_L measurement on data. In this case it

works because we are guaranteed the data should start in an eigenstate of Z_L. To show the errors induced by the transversal CNOT and to see the interaction between the data and the syndrome qubits, we compare to an experiment where we do not do the CNOT.

The experimental results for Steane-style EC are summarized in Table S2. Similar to Shor EC, feedback from ancilla introduces extra errors beyond the disturbance due to the EC circuit. We also postselect the experiment data with non-trivial (i) ancilla syndrome, (ii) data syndrome, or (iii) joint syndrome. We found that in the experiments with transversal CNOT applied, the rejection rate of (i) + (ii) exceeds that of (iii), indicating that transversal CNOT gate does propagate X errors from data to ancilla. Note that (i) on $\text{CNOT}_L|0_L\rangle|0_L\rangle$ is exactly the Steane-style state-preparation protocol, in which fault tolerance of the initial $|0_L\rangle$ states are often not assumed. The Z_L operator on ancilla can be served as an extra check for decreasing LER of postselected $|0_L\rangle$ state. As our direct preparation of $|0_L\rangle$ is already fault tolerant, we do not expect that Steane state preparation will significantly outperform the unitary protocol. The simulation results for $\text{CNOT}_L|0_L\rangle|+_L\rangle$ and $\text{CNOT}_L|0_L\rangle|0_L\rangle$ can be found in Tables 1 and 2.

Logical Bell-State Preparation We also study the performance of logical Bell state $|0_L0_L\rangle + |1_L1_L\rangle$, which can be directly prepared using the circuit $\text{CNOT}_L|+_L\rangle|0_L\rangle$. We use the same set of control and target qubits as in the Steane EC experiment (see Figure **??**e). We measure all qubits in Z (X) basis to extract the logical ZZ (XX) outcome. We first decode the measurement outcome of each logical qubit separately, then check whether the logical parity flips. We do not perform correlated decoding over two logical blocks. For logical X readout, transversal $Y(-\pi/2)$ rotations are performed on all qubits before the measurement, and the majority vote is performed over the parity of each column. The experiment and simulation results are presented in Table S3. We find that logical XX readout is significantly worse than ZZ in experiment. For the optimistic error budget improvement we have considered, the improvement of logical XX

readout is also not as significant as ZZ. We believe that the readout fidelity of XX is limited by both under-rotation errors in the final single-qubit Y gates and idle dephasing.

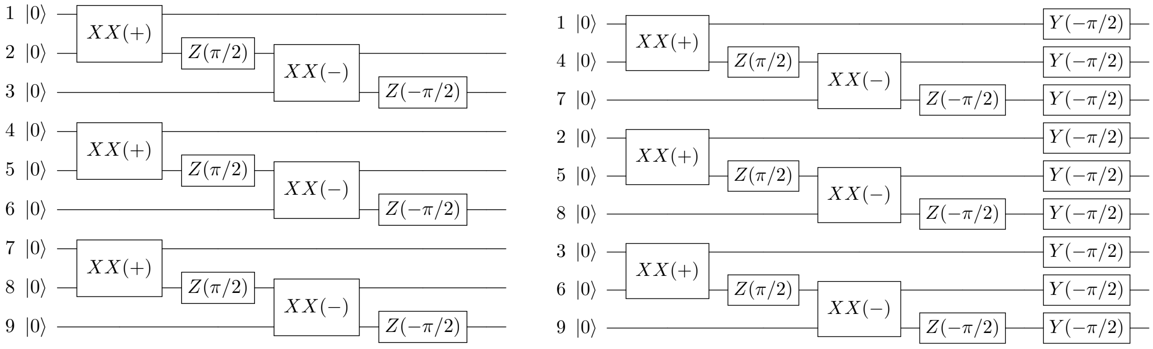

Figure S1: **Logical state preparation circuits using trapped-ion native operations.** Left: $|0\rangle$, Right: $|+\rangle$. $XX(\pm) = XX(\pm\pi/4)$.

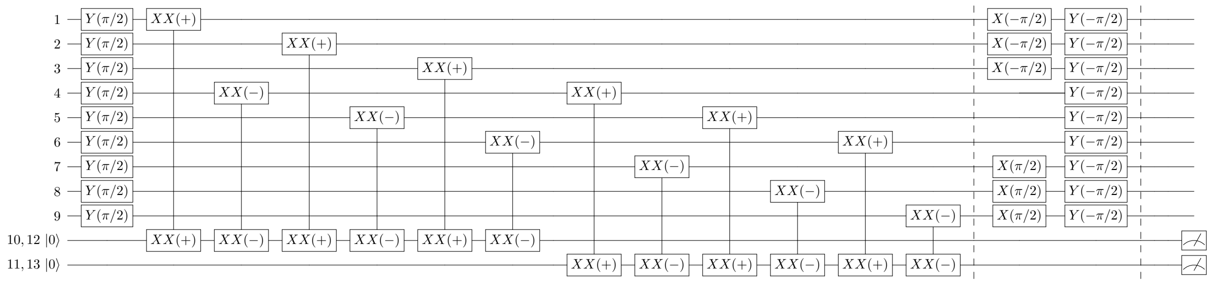

Figure S2: **Implementation of Shor-style syndrome extraction using trapped-ion native gates.** $XX(\pm) = XX(\pm\pi/4)$.

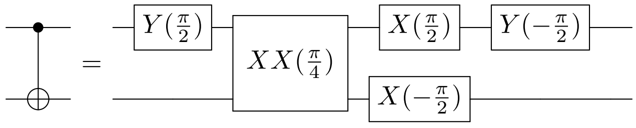

Figure S3: **Physical CNOT implementation using trapped-ion native operations.** In a transversal CNOT gate circuit, the physical CNOT circuits are applied sequentially on each data-ancilla pair.

$s^{(1)}$		00	10	11	01
μ_1	EXP	$64.68^{+1.88}_{-1.91}\%$	$10.40^{+1.26}_{-1.17}\%$	$7.20^{+1.08}_{-0.98}\%$	$17.72^{+1.55}_{-1.48}\%$
	SIM	$66.96^{+0.29}_{-0.29}\%$	$11.27^{+0.20}_{-0.20}\%$	$7.04^{+0.16}_{-0.16}\%$	$14.73^{+0.22}_{-0.22}\%$
	IMP	$79.29^{+0.25}_{-0.25}\%$	$6.44^{+0.15}_{-0.15}\%$	$3.87^{+0.12}_{-0.12}\%$	$10.40^{+0.19}_{-0.19}\%$
λ_1	EXP	$1.30^{+0.68}_{-0.49}\%$	$14.23^{+4.85}_{-4.01}\%$	$16.11^{+6.20}_{-5.05}\%$	$35.21^{+4.65}_{-4.45}\%$
	SIM	$0.82^{+0.07}_{-0.07}\%$	$10.71^{+0.59}_{-0.57}\%$	$17.02^{+0.90}_{-0.87}\%$	$33.38^{+0.77}_{-0.76}\%$
	IMP	$0.42^{+0.05}_{-0.04}\%$	$5.96^{+0.61}_{-0.57}\%$	$8.24^{+0.91}_{-0.85}\%$	$44.68^{+0.96}_{-0.96}\%$
δ_1	EXP	$1.30^{+0.68}_{-0.49}\%$	$6.92^{+3.80}_{-2.77}\%$	$20.00^{+6.60}_{-5.58}\%$	$9.71^{+3.15}_{-2.59}\%$
	SIM	$0.82^{+0.07}_{-0.07}\%$	$3.91^{+0.37}_{-0.35}\%$	$9.31^{+0.70}_{-0.67}\%$	$5.23^{+0.37}_{-0.35}\%$
	IMP	$0.42^{+0.05}_{-0.04}\%$	$3.43^{+0.47}_{-0.43}\%$	$9.64^{+0.97}_{-0.91}\%$	$5.44^{+0.45}_{-0.43}\%$
μ_2	EXP	$64.01^{+1.09}_{-1.10}\%$	$10.85^{+0.73}_{-0.70}\%$	$8.33^{+0.65}_{-0.62}\%$	$16.80^{+0.87}_{-0.84}\%$
	SIM	$67.01^{+0.29}_{-0.29}\%$	$10.95^{+0.20}_{-0.19}\%$	$6.92^{+0.16}_{-0.16}\%$	$15.12^{+0.22}_{-0.22}\%$
	IMP	$79.36^{+0.25}_{-0.25}\%$	$6.53^{+0.15}_{-0.15}\%$	$3.79^{+0.12}_{-0.12}\%$	$10.32^{+0.19}_{-0.19}\%$
λ_2	EXP	$13.31^{+0.99}_{-0.95}\%$	$25.18^{+3.13}_{-2.95}\%$	$30.08^{+3.76}_{-3.57}\%$	$24.60^{+2.48}_{-2.36}\%$
	SIM	$10.07^{+0.23}_{-0.23}\%$	$18.64^{+0.74}_{-0.73}\%$	$30.04^{+1.10}_{-1.08}\%$	$21.45^{+0.66}_{-0.65}\%$
	IMP	$6.14^{+0.17}_{-0.17}\%$	$9.69^{+0.74}_{-0.71}\%$	$19.46^{+1.30}_{-1.25}\%$	$13.60^{+0.68}_{-0.66}\%$
δ_2	EXP	$8.14^{+0.81}_{-0.76}\%$	$19.04^{+2.87}_{-2.64}\%$	$26.56^{+3.65}_{-3.42}\%$	$18.02^{+2.24}_{-2.09}\%$
	SIM	$4.49^{+0.16}_{-0.16}\%$	$11.66^{+0.62}_{-0.60}\%$	$19.78^{+0.96}_{-0.93}\%$	$13.98^{+0.56}_{-0.55}\%$
	IMP	$2.40^{+0.11}_{-0.11}\%$	$8.16^{+0.69}_{-0.65}\%$	$17.74^{+1.25}_{-1.20}\%$	$11.59^{+0.63}_{-0.61}\%$

Table S1: **The probability table for Shor-style syndrome extraction conditioned on the syndrome $s^{(1)}$ in the first round.** We calculate the probability of observing the first syndrome μ_k, the logical error rate λ_k with ancilla based correction, and the disturbance δ_k which is the logical error rate with only correcting on data measurement for $k = 1$ or $k = 2$ measurement of the stabilizer generators. We calculated these values based on the experimental data (EXP), our simulation of the experiment (SIM), and our predicted simulation for future experimental improvements (IMP).

Circuits	CNOT$_L\vert 0_L\rangle\vert +_L\rangle$	$\vert 0_L\rangle\vert +_L\rangle$	CNOT$_L\vert 0_L\rangle\vert 0_L\rangle$	$\vert 0_L\rangle\vert 0_L\rangle$	$\vert 0_L\rangle$ only
LER (without ancilla feedback)	$1.45^{+0.30}_{-0.26}\%$	$0.32^{+0.31}_{-0.18}\%$	$1.12^{+0.49}_{-0.37}\%$	$0.40^{+0.33}_{-0.21}\%$	$0.20^{+0.27}_{-0.14}\%$
LER (with ancilla feedback)	$4.93^{+0.51}_{-0.48}\%$	N/A	$3.20^{+0.53}_{-0.47}\%$	N/A	N/A
LER (with ancilla PS)	$0.66^{+0.27}_{-0.21}\%$		$0.34^{+0.33}_{-0.19}\%$		
RR (ancilla PS)	$(35.80 \pm 1.35)\%$	$(11.36 \pm 1.32)\%$	$(27.10 \pm 1.44)\%$	$(6.52 \pm 1.00)\%$	
LER (data PS)	$0.13^{+0.12}_{-0.07}\%$	$0.04^{+0.20}_{-0.04}\%$	$< 0.09\%$	$< 0.16\%$	$0.04^{+0.19}_{-0.04}\%$
RR (data PS)	$(17.19 \pm 0.94)\%$	$(6.36 \pm 0.99)\%$	$(14.44 \pm 1.05)\%$	$(6.88 \pm 1.03)\%$	$(5.08 \pm 0.88)\%$
LER (ancilla+data PS)	$0.05^{+0.21}_{-0.05}\%$	N/A	$< 0.11\%$	N/A	N/A
RR (ancilla+data PS)	$(42.57 \pm 1.48)\%$	$(16.88 \pm 1.61)\%$	$(32.68 \pm 1.58)\%$	$(12.28 \pm 1.37)\%$	

Table S2: **Experimental results of Steane-error correction (EC) and postselection (PS).** We consider two preparations of the logical ancilla qubit $|A_L\rangle$= $|0_L\rangle$ or $|A_L\rangle$=$|+_L\rangle$. Steane requires a logical CNOT and we compare experimental results when we apply it (CNOT$_L|0_L\rangle|A_L\rangle$) or not ($|0_L\rangle|A_L\rangle$). We also examine the experimental results when the ancilla qubit is not prepared ($|0_L\rangle$ only).

Logical operator		$Z_{L,1}Z_{L,2}$	$X_{L,1}X_{L,2}$
LER (EC)	EXP	$6.50^{+1.17}_{-1.04}\%$	$33.75^{+2.12}_{-2.07}\%$
	SIM	$(7.91 \pm 0.17)\%$	$(26.73 \pm 0.43)\%$
	IMP	$(1.25 \pm 0.07)\%$	$(18.96 \pm 0.24)\%$
LER (PS)	EXP	$0.58^{+0.61}_{-0.34}\%$	$16.13^{+3.22}_{-2.88}\%$
	SIM	$(1.02 \pm 0.08)\%$	$(7.05 \pm 0.53)\%$
	IMP	$< 10^{-4}$	$(2.01 \pm 0.16)\%$
RR (PS)	EXP	$(39.15 \pm 2.74)\%$	$(70.55 \pm 3.68)\%$
	SIM	$(37.77 \pm 0.38)\%$	$(77.41 \pm 0.86)\%$
	IMP	$(18.81 \pm 0.27)\%$	$(71.23 \pm 0.52)\%$

Table S3: **Experiment and simulation results for Bell-state preparation.** A logical error occurs when the measured joint logical parity is -1.
